# A Systematic Review of the Effectiveness of Non-Invasive Brain Stimulation Techniques to Reduce Violence Proneness by Interfering in Anger and Irritability

**DOI:** 10.3390/jcm9030882

**Published:** 2020-03-24

**Authors:** Ángel Romero-Martínez, Sara Bressanutti, Luis Moya-Albiol

**Affiliations:** Psychobiology Department, University of València, Blasco Ibañez Avenue 21, 46010 Valencia, Spain; sabres@alumnl.uv.es (S.B.); luis.moya@uv.es (L.M.-A.)

**Keywords:** anger, brain, magnetic stimulation, prefrontal cortex, violence

## Abstract

The field of neurocriminology has proposed several treatments (e.g., pharmacological, brain surgery, androgen-deprivation therapy, neurofeedback) to reduce violence proneness, but unfortunately, their effectiveness has been limited due to their side-effects. Therefore, it is necessary to explore alternative techniques to improve patients’ behavioural regulation with minimal undesirable effects. In this regard, non-invasive brain stimulation techniques, which are based on applying changing magnetic fields or electric currents to interfere with cortical excitability, have revealed their usefulness in alleviating the symptomatology of several mental disorders. However, to our knowledge, there are no reviews that assess whether these techniques are useful for reducing violence proneness. Therefore, we conducted a systematic review following Preferred Reporting Items for Systematic Reviews and Meta-Analyses (PRISMA) criteria using the following databases: PsycINFO, PubMed, Dialnet, Psicodoc, Web of Knowledge, and the Cochrane Library. We initially identified 3746 entries, and eventually included 56 publications. Most of the studies were unanimous in concluding that the application of these techniques over the prefrontal cortex (PFC) was not sufficient to promote anger and irritability reductions in euthymic individuals of both genders. Nevertheless, the application of non-invasive brain stimulation techniques, especially transcranial direct current stimulation, over the right PFC seemed to reduce violent reactions in these individuals by interfering with the interpretation of the unfavourable situations (e.g., threating signals) or inner states that evoked anger. In antisocial and pathological populations, the conclusions were provided by a few pilot studies with important methodological weaknesses. The main conclusion of these studies was that bilateral stimulation of the PFC satisfactorily reduced anger and irritability only in inmates, patients with autism spectrum disorders (ASD), people who suffered a closed-head injury, and agitated patients with Alzheimer’s disease. Moreover, combining these techniques with risperidone considerably reduced aggressiveness in these patients. Therefore, it is necessary to be cautious about the benefits of these techniques to control anger, due the methodological weaknesses of these studies. Nonetheless, they offer valuable opportunities to prevent violence by designing new treatments combining brain stimulation with current strategies, such as psychotherapy and psychopharmacology, in order to promote lasting changes.

## 1. Introduction

In recent decades, criminologists have paid attention to the growing knowledge that the neurosciences offer about human behavioural dysregulations. This situation led to the birth of neurocriminology. Neurocriminology is the scientific study of the biological bases (and their interactions with environmental variables) of violence proneness, as well as the application of this knowledge to prevent and/or reduce it [1,2,3]. A deeper understanding of the aetiology of violence will make it possible to design specific treatments and/or increase the effectiveness of current ones.

Even though several psychotherapeutic treatments have been proposed to reduce violence, their effectiveness has been limited [1,2,3]. In fact, a large number of subjects tend to reoffend after treatment. Moreover, many patients tend to abandon the intervention before it ends [4]. Thus, combining the above-mentioned treatments with pharmacological strategies, such as antidepressants, mood stabilizers, antipsychotics, and beta-blockers has been recommended and tends to have long-lasting effects [5,6]. Unfortunately, in many patients, these drugs tend to present side-effects, such as loss of sexual desire, weight gain, and insomnia, among others, during the initial stages of treatment [6,7,8]. These side-effects increase the risk of discontinuing the treatment before the appearance of therapeutic effects, which in turn increases the risk of violence recidivism and/or the maintenance of previous behavioural dysregulations. Furthermore, it is well-known that some of these violent individuals refuse to take the treatment because they do not recognize that they have mental health problems [9]. Finally, not all patients tend to respond to pharmacological strategies; there are even individuals who are refractory or intolerant to specific drugs. Moreover, it is necessary to maintain their use over long periods of time, which increases the risk of discontinuation. Thus, it is important to explore alternative therapeutic strategies in order to reduce aggressive behaviour.

In recent years, many researchers have employed non-invasive brain stimulation techniques to alleviate the symptomatology of several psychiatric disorders, such as depression, anxiety, obsessive-compulsive disorder, and bipolar disorder [10,11,12,13,14]. In particular, some, but not all, of these techniques are valid therapeutic alternatives for treatment-refractory major depression [15]. Additionally, these techniques also present diagnostic applications in neurology, such as presurgical motor and language mapping, and they are a valid instrument to diagnose several neurological conditions. These tools generally apply changing magnetic fields or electric currents through the surface of the skull to the neurons in the cortex. These changing magnetic pulses and/or current flows interfere with the depolarization of a group of neurons, which in turn affects their synapsis with other neurons transmitting these alterations in neural circuits and/or the brain networks [13,16,17]. Hence, we cannot consider their effect to be only local, because their initial effect tends to transynaptically extend across brain networks. Moreover, it has been suggested that their effects are capable of inducing changes in cortical excitability that can be maintained for days, but it is less clear how long these effects last after this period [17]. Moreover, it should be highlighted that cognitive processes and behaviour are sustained by complex neural networks made up of cortical and subcortical structures [5]. Therefore, the application of changing magnetic pulses and/or electric currents over the brain’s cortex might partly modulate these processes.

The most common and well-known non-invasive brain stimulation techniques are repetitive transcranial magnetic stimulation (rTMS), transcranial static magnetic field stimulation (tSMS), transcranial direct current stimulation (tDCS), and cranial electrotherapy stimulation (CES). Although these techniques are based on the effects of applying changing magnetic fields or electric currents to interfere in cortical excitability, they differ in several aspects. Thus, their effects vary, for example, depending on the use of a coil or electrodes, the coil shape, the stimulation site, the pattern of stimulation, polarity, and current intensity, among others [13,14,15,16,17,18,19]. For example, in TMS devices, an electric pulse is sent through the coil, which generates a changing magnetic pulse. When this coil is placed over the head, this magnetic pulse travels across the skull. These magnetic pulses tend to interfere in the depolarization of a group of neurons. It can be applied as single or repetitive pulses (repetitive TMS or rTMS). The Theta-Burst Stimulation (TBS) represents a patterned form of rTMS. In fact, patients receive a series of short magnetic pulses (bursts) at a high frequency, which corresponds to theta brain oscillations. These bursts can be applied as continuous (cTBS) or intermittent protocols (iTBS). Regarding the tSMS, it is based on the principle of applying static magnetic fields over the scalp with a constant intensity and orientation, which tends to reduce cortical excitability. With regard to tDCS, it consists of the application of weak and constant direct current to the brain via electrodes placed on the head. These current flows modulate cortical excitability, which would lead to facilitation of neural excitation (anodal tDCS) or inhibition of neural activity (cathodal tDCS). Lastly, CES devices generate and send low-intensity alternating current electrical stimulation via electrodes that can be placed, for example, on the earlobes [13,14,15,16,17,18,19].

Violence in humans is a complex phenomenon with multiple explanatory causes. Many authors have identified two specific cortical structures, the prefrontal cortex (PFC) and the temporal lobe, as important for behavioural regulation. In this regard, violence proneness is commonly present after registering injuries (e.g., tumours, traumatic brain injuries, brain haemorrhages) in these cortical structures [3,4,5]. In fact, specific traumas in one or both of these brain structures might facilitate outbursts and violent reactions due to difficulties in inhibiting limbic irritability [3,4,5]. This is a well-known model to explain reactive violence or the violence guided by emotional insights after perceiving a potential threat. Nevertheless, there is another kind of violence, known as proactive, that is characterized by predatory unemotional attacks rather than emotional reactions. Because these two types of reactions are explained by different cognitive processes, it is logical to conclude that their underlying brain structures would differ. Proactive reactions tend to be related to an increased activation of the ventral striatal and the angular gyrus [5]. Nonetheless, it is not well-understood whether non-invasive brain stimulation techniques would be appropriate to reduce specific behavioural dysregulations, such as violence proneness, by interfering in current brain networks by applying magnetic pulses and/or current flows. Thus, it would be interesting to conduct a systematic review assessing whether these techniques, without controlling for coadjutant treatments, are appropriate for decreasing violence proneness.

With all this in mind, the principal aim of this systematic review was to answer the question of whether specific non-invasive stimulation techniques (i.e., rTMS, TBS, tSMS, tDCS, and CES) are valid strategies on their own to reduce anger, hostility, and/or irritability levels (e.g., state or trait), thus decreasing violence proneness. This would occur because violence is partly explained by alterations in anger-states, which are part of the violence facilitation system [20,21]. Therefore, our first aim was to analyse whether studies registered changes in several facets of human violence, such as feelings of anger and anger expression, after the application of these non-invasive brain stimulation techniques in several populations (normative, violent individuals, and patients with mental disorders/pathological conditions). Moreover, several variables would be considered, such as demographic variables (e.g., age, gender), the brain structures stimulated, and variables of the non-invasive brain stimulation techniques (e.g., intensity, allocation, number of sessions), as potential mediators in the effects of these techniques on violence proneness. Finally, considering the existing data so far, we propose a series of recommendations for the correct application of non-invasive brain stimulation techniques to reduce violence proneness. Moreover, the conclusions derived from this manuscript will help the scientific community, clinicians, and patients to learn about the available evidence on treatments for violence control and their advantages and disadvantages in order to make evidence-based choices.

## 2. Search Strategy

We followed the Preferred Reporting Items for Systematic Reviews and Meta-Analyses (PRISMA) quality criteria for reviews to conduct this systematic review [5,6,22,23]. A literature search was performed through the following databases: PsycINFO, PubMed, Dialnet, Psicodoc, Web of Knowledge, and the Cochrane Library. Moreover, we also completed the previously mentioned process with hand-searching. All these processes were carried out from October to December of 2019. Regarding manuscript selection, we paid special attention to carefully choosing manuscripts with good methodological quality in order to increase the value of this systematic review. Nevertheless, some manuscripts were selected due to the lack of scientific literature in this specific field (e.g., inmates, pathological conditions).

The search strings considered relevant for this field of research and applied to the databases were: ((Transcranial magnetic stimulation) OR (transcranial direct current stimulation), OR (cranial electrotherapy stimulation) OR (theta burst stimulation), OR (transcranial static magnetic field stimulation)) AND ((rumination) OR (mood) OR (violence) OR (aggressive) OR (anger) OR (hostility)) OR (irritability) OR (inmate)).

All the papers selected for final inclusion met the following criteria: (a) they were empirical studies with humans; (b) they assessed the association of non-invasive brain stimulation technique application with trait or state anger, proneness to violence, and aggressive strategies in laboratory tasks; (c) there was no controlled concomitant psychotropic medication treatment or statistical control of their role during TMS treatment; (d) presence of a control group (e.g., sham-controlled, waiting list) and/or randomization of the sample; (e) they did not collapse non-invasive brain stimulation techniques without differentiating the effects of each; and (f) they were written in English. If we found a manuscript that did not offer much information about inclusion/exclusion criteria or participants’ characteristics, we decided to discard the manuscript. For example, several manuscripts included pathological conditions closely related to irritability (e.g., Alzheimer Disease, epilepsy, autism spectrum disorders), but we only included those that did not mix, or controlled for, psychotropic treatments with non-invasive brain stimulation techniques.

Article selection was carried out by two independent researchers. The level of interrater agreement between the two researchers was 95%. In cases of disagreement, we discussed these manuscripts in order to see whether they adhered to the inclusion criteria of our systematic review.

## 3. Results

**Figure 1:**
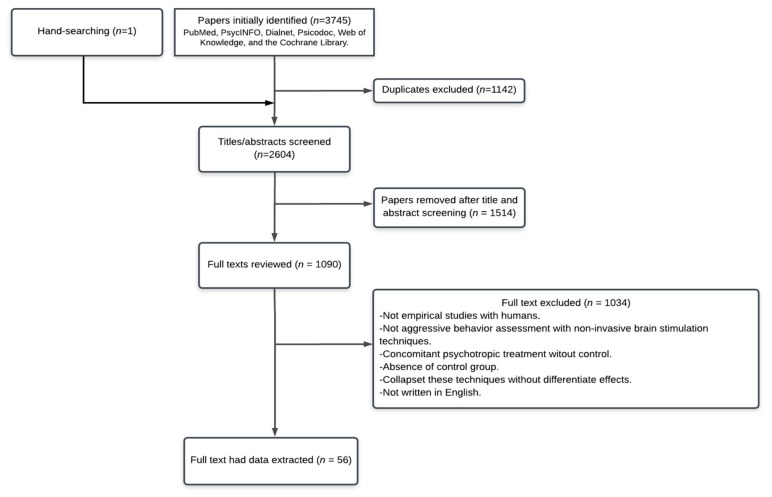
Flow chart of literature search with reasons for exclusion.

We initially identified 3745 publications in PsycINFO, PubMed, Dialnet, Psicodoc, Web of Knowledge, and the Cochrane Library. Furthermore, we included an additional reference found by hand-searching that was not included in the previously mentioned databases. After assessing the existence of duplicated manuscripts, 1142 were removed, leaving 2604 for the screening of titles and abstracts. After that, the full text of 1090 articles were read, finally including 56 publications (Figure 1). The main characteristics of the participants and studies included in this review are summarized in Table 1 (e.g., participants’ characteristics, brain structures stimulated, main results of anger-state assessment, type of design).

Initially, due to the number of studies included with a normative population, we decided to divide them into two blocks. First, we summarized studies that analysed whether brain stimulation techniques produced anger state (measured by self-reports) improvements and/or changes. Second, manuscripts were presented that assessed how brain stimulation interferes with participants’ performance on laboratory tasks that assess violence. Nonetheless, it should be noted that certain studies were repeated in both blocks because they studied mood changes assessed by self-reports and laboratory task performance. Afterwards, we will present the results based on a study that applied these techniques in violent offenders—specifically, inmates. Moreover, we finish the results by presenting several studies with patients affected by autism spectrum disorder (ASD), unipolar depressed patients of the melancholic subtype, abstinent smokers, people with language disorders, closed-head-injury patients, Alzheimer’s, refractory partial epilepsy and Schizophrenia.

### 3.1. Normative Individuals

By including studies with normative participants, we increased the external validity of the conclusions derived from our study. In fact, this will reveal whether these techniques would be useful to promote mood changes not only in individuals with pathological conditions, but also in healthy individuals.

### 3.2. Self-Reports

Surprisingly, only three studies [28,53,78] out of 35 (0.9%) revealed significant reductions in anger state after the application of brain stimulation techniques. It is interesting to highlight that both studies mainly stimulated the prefrontal cortex (PFC), which has usually been associated with behavioural control [79]. Nevertheless, most of the studies that also stimulated the PFC failed to report significant anger-state or irritability improvements after the application of these techniques [24,25,26,27,29,30,31,32,33,34,35,37,38,39,40,41,42,43,44,45,46,48,49,50,51,52,54,57]. The rest of the studies focused the brain stimulation on the left temporo-parietal junction [36], vermis and cerebellar hemispheres [56], inferior frontal cortex [47], and earlobes [55], but they also failed to reduce anger. These studies seem quite unanimous in concluding that there are no effects on mood in normative populations. In order to understand these results, it is important to pay attention to the methodological aspects of the above-mentioned studies. For example, there were several brain stimulation techniques employed that stimulated different brain regions, with different intensities or frequencies of brain stimulation and patterns of application (e.g., single session, several sessions over several weeks, consecutive or alternating sessions). Furthermore, most of them included sham-controlled brain stimulation as a control condition. It is important to know that all of them were based on healthy young adults around 20 years old, which increases the reliability of the results.

Conversely, the two studies that revealed significant results presented important methodological weaknesses that should be mentioned. In this regard, Hoffman et al. [28] only employed 20 participants with a pre-post design without a placebo group (sham-controlled). Additionally, Choy et al. [53] assessed violence tendencies based on hypothetical scenarios that participants had to rate on a quantitative continuous scale (from 0 to 100). Although the sample size was relatively high in comparison with the previous study, the use of a test without providing internal consistency and reliability reduced our confidence in these results. The third study failed to obtain significant reductions in irritability on one of the self-reports included to assess irritability [29]. Finally, based on these limited sample sizes and the high number of statistical comparisons, it would be necessary to conduct Bonferroni corrections for multiple comparisons in both studies. However, the authors did not control or report this aspect. Therefore, we assumed that these techniques were not sufficient to promote anger state changes in normative healthy young adults.

### 3.3. Laboratory Task

With regard to violence proneness measured by laboratory tasks, most of the studies included revealed significant and relatively similar results. Furthermore, it is interesting to note that these studies measured violence proneness with a well-validated computer task, the Taylor Aggression Paradigm (TAP) [41,47,48,54,61,62,63,64]. On this task, participants compete with a virtual opponent, and they must respond to a provocation (fictitious character/opponent). In fact, they can respond and punish the opponent with an electric shock. This test makes it possible to manipulate the degree of provocation and assess the participants’ perception of provocation and their type of aggressive reaction (reactive or proactive). Finally, it is also important to keep in mind that all the studies included a sham-group as controls, and some of them were double-blinded [41,54,60,63].

Together, the studies paid attention to the effects of brain stimulation on the PFC in interfering with participants’ aggressive behaviour measured by the TAP. First, when participants received stimulation over the right dorsolateral prefrontal cortex (DLPFC), only men experienced a reduction in proactive violence on the TAP [61]. Conversely, the stimulation of the left DLPFC increased reactive and proactive violence in both genders after provocation [59], but a study with women failed to obtain differences across conditions [65]. Likewise, another study revealed that complete stimulation of the left frontal cortex also increases aggressive reactions to provocation [41]. If we focus on other regions of the PFC, the results are consistent with those mentioned above. In other words, stimulation of the right PFC entails decreases in aggressive behaviour, whereas stimulation of the left PFC increases aggressive behaviour. Thus, after stimulation of the right ventrolateral prefrontal cortex (VLPFC), participants experienced a decrease in violent reactions to provocation [64], even after being exposed to violent videogames [24,27,37,46,47,48,52,55,56,57,58,61,62,63,64]. Nonetheless, stimulation of the left VLPFC increased aggression after provocation [54]. Furthermore, bilateral stimulation of the ventromedial PFC (VMPFC) produced a decrease in their aggressive reactions after provocation [63]. Moreover, stimulation of the right inferior frontal gyrus was also associated with less explicit violent reactions to provocations but showed a Machiavellian reaction to increase their chances of obtaining profits [60]. Lastly, contrary to these conclusions, only one study that bilaterally stimulated the inferior frontal cortex presented no effect on TAP performance [48].

The studies included in this block presented relatively homogeneous methodological characteristics (sample size, sample demographic characteristics, anger assessment, study design). This increased the reliability of the results but reduced the external validity of the conclusions. Moreover, it should be highlighted that most of the studies reported significant differences, but most of these studies divided the sample into small groups across conditions. Having a reduced sample size that was subsequently divided into additional groups increased the likelihood of a type I error. Thus, most of these significant differences were close to 0.05. Thus, if they did not correct for multiple comparisons, this reduced our confidence in these results. It should also be highlighted that these studies only paid attention to PFC, and neglected other cortical regions closely related to aggression proneness.

### 3.4. Violent Population

To extend the external validity and clinical applications of these results, it is necessary to include violent individuals. As explained above, we only included manuscripts that excluded participants with concomitant psychotropic medication or that statistically controlled their effects. Based on this criterion, we only included six manuscripts. First, we will describe the results of a manuscript that studied brain stimulation techniques with inmates. After that, we will provide information about five studies with patients with autism spectrum disorders (ASD).

A single study focused on the effects of non-invasive brain stimulation techniques on behavioural dysregulation in inmates [66]. It is important to keep in mind that this study included a sham-controlled group, but with a reduced sample size (less than 10 participants per group). The authors observed that inmates, especially the group of murderers, experienced a decrease in self-reported anger expression (physical and verbal) after bilateral anodal tDCS of the PFC. Despite these encouraging results, the group of murderers was too small (eight participants). Furthermore, it did not control for potential confounding variables. In fact, prison populations are commonly heterogeneous in their demographic characteristics, but the authors did not offer much information about each group in order to understand how these variables affected participants’ sensitivity to brain stimulation effects. Lastly, the authors commented that they included a non-prisoner group as controls, but they did not offer information about this group (sample size, characteristics, sham-controlled or not).

### 3.5. Pathological Conditions

Regarding studies with patients with ASD, all of them reported improvements in behavioural regulation (e.g., irritability, agitation, repetitive behavioural patterns) after bilateral stimulation of the DLPFC [67,68,69,70,71]. Even though these results are encouraging regarding the effects of rTMS in this population, these studies presented several methodological limitations that should be discussed. For example, the main limitation is the type of research design, without double-blinding and randomization of the sample. Another important limitation is the reduced sample size, with no more than 60 participants in each study. Furthermore, these studies included half of the ASD participants as a waiting-list control group, but this is not adequate as a control. It would be necessary to include a sham-controlled group, or even a third group of unaffected participants. Hence, this diminishes the confidence in these results and reinforces the need to conduct additional studies with robust research designs. There were also two studies that showed significant reductions in anger levels in two different populations, such as patients who had suffered a closed-head injury 6 months earlier [75] and Alzheimer patients [76]. Finally, other studies reported a lack of effects on treatment-resistant depressive patients with no pharmacological treatment [72], on anger-state in current-abstinent smokers [73], on patients with refractory partial epilepsy who received a dosage of antiepileptics [77], and on patients with schizophrenia [78]. Even a study with mentally retarded minors with language disorders reinforced the fact that non-invasive brain stimulation techniques produced an increase in irritability in approximately 40% of these patients [74]. Curiously, these studies presented more methodological strengths than the aforementioned studies with positive changes. Thus, this fact should be discussed properly in order to clarify these unknowns.

## 4. Discussion

To our knowledge, there is no other literature review that provides a detailed description of the effects of non-invasive brain stimulation techniques on anger management in several populations. We carefully selected studies with good methodological quality (e.g., double-blind, randomized, sham-controlled). Most of the studies were unanimous in concluding that non-invasive brain stimulation techniques on the PFC is not sufficient to promote mood alterations (e.g., anger-state) in euthymic (normative) populations of both genders. Nevertheless, the dominance of the right PFC over the left PFC reduced violent reactions in normative individuals of both genders by interfering in the interpretation of the unfavourable situations or inner states that evoked anger. In antisocial individuals and people with mental disorders, bilateral stimulation of the PFC satisfactorily reduced anger and irritability in inmates, patients with autism spectrum disorders (ASD), patients who had suffered a closed-head injury, and agitated patients with Alzheimer’s disease. Moreover, combining these techniques with risperidone considerably reduced aggressiveness in these patients. Unfortunately, in people with language disorders, people with treatment-resistant depression, smokers, and others with refractory partial epilepsy and schizophrenia, it did not promote anger-state alterations. Therefore, it is necessary to be cautious about the benefits of these techniques for controlling anger and irritability, and it is relevant to consider these strategies to reduce anger-state as coadjutant treatments to psychotherapy and psychopharmacology in order to promote lasting changes in violent populations.

The studies included in this review indicate that there is an asymmetric predominance of PFC functioning in eliciting anger states, lowering the threshold of their appearance when activation of the left PFC predominates over the right, specifically, stimulation of the left DLPFC and VLPFC. By contrast, the activation of the right DLPFC, VLPFC, and inferior frontal gyrus, as well as the bilateral VMPFC, tends to be associated with decreases in anger-oriented states and increased efforts to control anger expression [41,47,48,54,59,60,61,62,63,64]. Obviously, the relationship between the application of non-invasive brain stimulation techniques over the PFC and the reduction in anger or irritability is complex, as expected. Moreover, it is also important to keep in mind that the PFC is not only involved in anger induction [20]. Mood induction is sustained by several brain circuits, including cortical and subcortical structures [5]. Thus, it would be possible to interfere in the threshold of the appearance of violence by interfering in the interpretation of contextual or inner states, instead of modifying mood.

Regarding mood alterations, studies were unanimous in sustaining that, in euthymic (normative) individuals, we cannot conclude that non-invasive stimulation techniques might promote mood improvements, particularly decreases in anger, but it seemed relevant for murderers [60], young individuals with ASD [67,68,69,70,71], patients who had suffered a closed-head injury [75], and agitated patients with Alzheimer’s disease [76]. It is important to note that pathological populations might be sensitive to brain stimulation because their functioning is relatively different from the normative. For example, it has been demonstrated that antisocial individuals show increased left PFC predominance over the right PFC [80]. This hemispheric dominance might explain the frequent appearance of an approach-oriented emotion of anger in response to a minimal sign of threat or the maintenance of these emotional states as a characteristic of an individual (high anger trait). Therefore, it makes sense that the bilateral application of non-invasive brain stimulation techniques over the DLPFC might help to regulate hemispheric asymmetries in violent inmates or ASD patients. Nevertheless, it was not useful for individuals with language disorders, treatment-resistant depressive patients without antidepressants, and recently abstinent smokers. Thus, it is difficult to conclude that this technique was useful for all types of patients, without considering coadjutant pharmacological treatments. In fact, approximately half of the mentally retarded with language disorders presented an increase in irritability after several sessions of tDCS [74]. This is relevant because these participants were minors, which tends to be a sensitive population to pharmacological treatments [81].

With regard to the characteristics of the non-invasive brain stimulation techniques, for tDCS, the application of 1 to 2 mA for 15–30 min seems sufficient to promote the aforementioned alterations in the interpretation of hostile intentions of our opponents. For cTBS and rTMS, it is sufficient to apply 0.5 to 1 Hz for 30 min. Nevertheless, CES did not seem to promote these mood changes. This could be explained by its far location from the PFC. Fortunately, we also consider it important to highlight that none of the studies reported important side-effects, only unusual minimal reactions. Finally, because the effects of these stimulation techniques seem relatively short-lived, it would be advisable to sustain them with single weekly sessions spaced out over time. In this regard, there was no unanimity among the studies included in our review about whether a single session is sufficient to produce changes, or whether several sessions are required. Therefore, it is necessary for further studies to explore this key variable.

Some strengths of this systematic review should be highlighted. First, we considered several multidisciplinary scientific databases. Moreover, it is also important to acknowledge that we considered grey literature and clinical trials, which increased the likelihood of obtaining unpublished research. Second, it is extremely important to reinforce the fact that we established the methodological quality of the manuscripts as an inclusion criterion, thus excluding case studies or weak research designs. Finally, our conclusions were based on several studies with relatively homogeneous samples, which positively impacts the replicability of the results and reinforces the value of our conclusions.

This article also had a few limitations that are important to mention and consider in the interpretation of the results. Because we only searched for studies in specific databases, other databases were omitted that could have increased the range of articles found. Furthermore, there was an unbalanced number of manuscripts, predominantly including studies assessing anger and irritability in normative individuals, but only a few measured aggressive behaviours in violent individuals (e.g., people with mental disorders, inmates, offenders). However, studies assessing whether these techniques are good enough to promote changes had important methodological limitations. This reduced the reliability of our conclusions, especially in those analysing violent and pathological populations, thus reinforcing the need to conduct additional research in this field. Moreover, a large number of studies assessed anger with self-reports, which are subject to distortions such as social desirability and acquiescence biases. Additionally, criminal offenders and inmates might present a conflict of interest on questionnaire responses due to the obtaining of penitentiary benefits. Regarding TAP, although it is a well-known laboratory procedure, it only assesses aggressive behaviour in response to provocation. Therefore, it should be noted as a potential limitation. Furthermore, several manuscripts combined psychotropics with brain stimulation techniques, and so it is difficult to assess the effects of brain stimulation techniques alone. In fact, in these cases, it was very difficult to determine whether therapeutic effects were the result of each technique, or a combination of the two. Moreover, several studies did not offer information about whether patients had taken drugs, even though these patients presented with uncontrollable agitation and irritability. Because this sounded odd, we decided to remove them from our review. Nevertheless, we do not think this affected the conclusions of our study.

## 5. Conclusions

In summary, the present review demonstrated the importance of considering non-invasive brain stimulation techniques as potential tools to increase the threshold for becoming violent by interfering in the interpretation of threat signals. Moreover, it is important to highlight that we should consider the possibility of combining these treatments with other previously established treatments in order to increase their success. In any case, although these tools seem to be relevant strategies to reduce anger-state, we cannot ignore the study limitations and the need to consider it as a coadjutant treatment to psychotherapy in order to promote lasting changes in violent populations. Future studies should compare the effects of these tools combined with other pharmacological treatments (e.g., sertraline, impipramine, fluoxetine, risperidone) and serotonin norepinephrine reuptake inhibitors, such as venlafaxine, which not only affect the sertraline system, but also the noradrenergic system in order to find out whether other drugs with potent effects on other neurotransmitter systems present greater benefits for anger control.

## Figures and Tables

**Table 1:** Main sociodemographic characteristics and details about the participants in each study, the main results, and the assessment methods used.

Authors	Sample	Age, Gender and Handedness	Brain Structures	Brain Stimulation	Research Design	Main Results (Anger)
**Normative and healthy sample**						
**Self-reports**						
Schutter et al., [24]	12	28.4 ± 8.90	Right DLPFC	rTMS 1 Hz 20 min	Single blind, sham-controlled	STAS, absence differences
		67% men and 33% women		Single session		
		Right-handed				
Grisaru et al., [25]	18	40.5 ± 11.60	Left and right PFC	rTMS 1 Hz	Randomized, sham-controlled	VAS, absence differences
		39% men and 61% women		Four days		
		Right-handed				
Jenkins et al., [26]	19	24.6 ± 5.30	Left and right DLPFC	rTMS 1 Hz Two sessions spaced 2 weeks	Pseudo-randomization	POMS, absence differences
		47% men and 53% women				
		-				
Schutter et al., [27]	12	From 18 to 25	Left OFC	rTMS 1 Hz 20 min	Double-blind, sham-controlled	POMS and VAS, absence differences
		50% men and 50% women		Single session		
		Right-handed				
Hofman et al., [28]	20	21.0 ± 1.81	Right frontal cortex	rTMS 0.18 ± 0.02 Hz Single session	Pre-post design	Higher left-to-right transcallosal inhibition associated higher AQ score
		10% men and 90% women				
		Right-handed				
Schaller et al., [29]	38	24.0 ± 2.77	Left DLPFC	rTMS	Double-blind, sham-controlled	BDI, decrease in irritability
		100% men		25 Hz		VAS, absence differences
		Right-handed		9 sessions/consec. days		
Baeken et al., [30]	28	24.68 ± 5.85	Left DLPFC	rTMS 10 Hz 20 min Single session	Single-blind, sham-controlled	POMS and VAS, absence differences
		100% women				
		Right-handed				
Baeken et al., [31]	27	25.2 ± 5.00	Left DLPFC	rTMS 10 Hz 20 min Single session	Single-blind, sham-controlled	POMS and VAS, absence differences
		100% women				
		Right-handed				
Koenigs et al., [32]	21	25.6 ± 5.8	Bilateral frontal cortex	tDCS anodal and cathodal 2.5 mA 35 min	Double-blind, sham-controlled	POMS, absence differences
		57% men and 43% women		Single session		
		-				
Leyman et al., [33]	18	21.1 ± 1.45	Left and right DLPFC	rTMS 10 Hz Two sessions, spaced 1 week	Single-blind, sham-controlled	VAS, absence differences
		100% women				
		Right-handed				
Baeken et al., [34]	20	23.30 ± 2.94	Left and right DLPFC	rTMS 10 Hz 20 min	Single-blind, randomized	POMS, absence differences
		100% women		Single session		
		-				
Baeken et al., [35]	24	22.29 ± 2.58	Right DLPFC	rTMS 10 Hz 20 min	Single-blind, sham-controlled	Self-reported anger, absence differences
		100% women		Single session		
		Right-handed				
Baeken et al., [36]	36	21.20 ± 1.44	Right and left DLPFC	rTMS 10 Hz 20 min	Single-blind, randomized, sham-controlled	Self-reported anger, absence differences
		100% women		Single session		
		Right-handed				
Baumgartner et al., [37]	36	24.3 ± 4.2	Left temporo-parietal junction	rTMS 1 Hz 20 min Single session	Randomized, sham-controlled	Self-reported anger
		100% men				
		Right-handed				
Baeken et al., [38]	30	1.53 ± 2.85	Left DLPFC	rTMS 20 Hz 20 min Single session	Single-blind, sham-controlled	Self-reported anger, not mediate changes
		100% women				
		Right-handed				
Moulier et al., [39]	20	33.7 ± 12.2	Left DLPFC	rTMS 10 Hz 10 sessions/15 min/2 weeks	Double blind, Sham-controlled	VAS, absence differences
		60% men and 40% women				
		Right-handed				
Iyer et al., [40]	103	37.5 ± 12.9	Left PFC	tDCS anodal and cathodal 1–2 mA 20 min	Single-blind, sham-controlled	VAS, absence differences
		46% men and 54% women		Single session		
		Right-handed				
Hortensius et al., [41]	80	-	Frontal cortex	tDCS 2 mA 15 min Single session	Double blind, randomized, sham-controlled	Self-reported anger, absence differences
		50% men and 50% women Right-handed				
Plazier et al., [42]	17	21.47 ± 0.91	Right (anodal) and left (cathodal) DLPFC and occipital	tDCS 1.5 mA 20 min	Double blind, randomized, sham-controlled	Self-reported anger, absence differences
		100% men		Single session		
		-				
Motohashi et al., [43]	12	22 ± 2.2	Left DLPFC	tDCS 1 mA 4-daily 20 min	Single-blind, sham-controlled	POMS, absence differences
		100% men		Four days		
		83% right-handed				
Kelley et al., [44]	90	-	Left and right PFC	tDCS 2 mA 15 min Single session	Double-blind, sham-controlled	Self-reported anger, absence differences
		33% men and 67% women				
		Right-handed				
McIntire et al., [45]	30	29.3 ± 3.4	DLPFC	tDCS (anodal) + caffeine 2 mA 30 min	Randomized, sham-controlled	POMS and VAS, absence differences
		73% men and 26% women		Single session		
		Right-handed				
Vitor-Costa et al., [46]	11	26 ± 4	Primary motor cortex	tDCS 2 mA 30 min Three days, spaced 48 h	Single-blind, sham-controlled	Self-reported anger, not mediate changes
		100% men				
		-				
Riva et al., [47]	80	23.06 ± 4.36	Right VLPFC	tDCS 1.5 mA 20 min Single session	Randomized, sham-controlled	STAS, absence differences
		21% men and 79% women				
		-				
Dambacher et al., [48]	64	21.89 ± 3.26	Inferior frontal cortex	tDCS 1–2 mA 21.75 min	Randomized, sham-controlled	RPQ, absence differences
		61% men and 39% women		Single session		
		-				
De Putter et al., [49]	66	23.09 ± 5.03	DLPFC	tDCS 2 mA 25 min	Double blind, Sham-controlled	POMS, absence differences
		20% men and 80% women		Single session		
		-				
De Raedt et al., [50]	32	22.6 ± 2.3	DLPFC	tDCS (anodal) 1.5 mA 20 min	Single-blind, sham-controlled	STAS, absence differences
		100% women		Single session		
		Right-handed				
McIntire et al., [51]	50	27 ± 5	Left (anodal) and right (cathodal) DLPFC	tDCS + caffeine 2 mA	Random, sham-controlled	POMS, absence differences
		72% men and 28% women		36 h		
		-				
Vanderhasselt et al., [52]	35	23.40 ± 4.43	Right DLPFC	tDCS (anodal) 2 mA 20 min	Single-blind, sham-controlled	VAS, absence differences
		31% men and 69% women		Single session		
		Right-handed				
Choy et al., [53]	81	20 years	Bilateral DLPFC	tDCS (anodal) 2 mA 20 min	Double-Blind, Placebo-Controlled, Stratified, Parallel-Group Trial	Increases activation PFC less desire to commit physical and sexual assault (hypothetical vignettes/scenarios)
		44% men and 56% women		Two sessions		
		-				
Gallucci et al., [54]	90	22.27 ± 2.46	VLPFC	tDCS (anodal) 1.5 mA 20 min	Double blind, randomized placebo-controlled design; sham-controlled	STAS, absence differences
		50% men and 50% women		Single session		
		-				
Valenzuela et al., [55]	8	27 ± 2	Left primary motor cortex	tDCS (anodal) 2 mA 20 min	Double-blind, cross-over, sham-controlled	BMS, absence differences
		100% males		Single session		
		-				
Roh et al., [56]	50	54.8 ± 2.8	Earlobes of patients	CES 0.5 Hz 20 min 3 times/week; 8 weeks	Cross-over, sham-controlled	POMS, absence differences
		100% women				
		-				
Demirtas-Tatlidede et al., [57]	12	28.8 ± 9.94	Vermis and cerebellar hemispheres	iTBS 10 burst/session Three sessions	Randomized	POMS, absence differences
		50% men and 50% women				
		Right-handed				
Sheffield et al., [58]	24	26.54 ± 12.28	Left or right PFC (frontal alpha asymmetry)	tSMS Single session	Double-blind, sham-controlled	AQ, absence relationship cortical changes and AQ score
		54% men and 46% women				
		Right-handed				
**Laboratory tasks**						
Perach-Barzilay et al., [59]	16	28 ± 4.68	Left DLPFC	cTBS 5 Hz/50 bursts Single session	Randomized placebo-controlled design; sham-controlled	SOP, stimulation left DLPFC increased reactive and proactive aggression
		88% men and 12% women Right-handed				
De Dreu et al., [60]	18	25.16 ± 2.00	Right inferior frontal gyrus	TBS Three sessions	Double-blind, sham-controlled	TAP, High activation entailed less aggression
		100% men				
		-				
Hortensius et al., [41]	80	-	Frontal cortex	tDCS 2 mA 15 min Single session	Double blind, randomized placebo-controlled design; sham-controlled	TAP, left frontal activity entailed high aggression after provocation
		50% men and 50% women Right-handed				
Riva et al., [47]	80	23.06 ± 4.36	Right VLPFC	tDCS 1.5 mA 20 min Single session	Randomized, sham-controlled	TAP, Anodal stimulation right VLPFC entailed less aggression in socially excluded participants after videogame exposure
		21% men and 79% women				
		-				
Dambacher et al., [48]	64	21.89 ± 3.26	Bilateral inferior frontal cortex	tDCS 1.5 mA 21.75 min Single session	Randomized, sham-controlled	TAP, absence differences
		61% men and 39% women				
		-				
Dambacher et al., [61]	43	22.14 ± 2.00	Right DLPFC	tDCS 2 mA (20 phases) 750 s	Randomized placebo-controlled design sham-controlled	TAP, right hemispheric dominance reduced proactive aggression in men
		47% men and 53% women		Single session		
		-				
Riva et al., [62]	79	21.73 ± 2.38	Right VLPFC	tDCS (anodal) 1.5 mA 20 min	Randomized placebo-controlled design; sham-controlled	TAP, Lower levels of aggressive behaviour
		52% men and 48% women		Single session		
		-				
Gilam et al., [63]	25	26.16 ± 3.63	Bilateral VMPFC	tDCS (anodal)	Double-blind, sham-controlled	Increased activation entailed less self-reported anger after provocation
		40% men and 60% women		1.2 mA 22 min Two sessions		
		-				
Chen et al., [64]	32	20–22 years	Right VLPFC	tDCS 2 mA 20 s	Randomized, sham-controlled	TAP, Reduction in proactive and reactive aggression
		50% men and 50% women		Single session		
		-				
Gallucci et al., [54]	90	22.27 ± 2.46	Left VLPFC	tDCS (anodal) 1.5 mA 20 min	Double-blind, randomized sham-controlled	Left VLPFC increased aggression.
		50% men and 50% women		Single session		Males were more aggressive than females
		-				
Dedoncker et al., [65]	41	22.9 ± 2.61	Left DLPFC	tDCS (anodal) 1.5 mA 20 min	Randomized sham-controlled	VAS, absence changes
		100% females		Single session		
		Right-handed				
**Violent individuals (inmates)**						
Molero-Chamizo et al., [66]	41	36.2 ± 12.3	Bilateral PFC	tDCS (anodal)	Single-blind, sham-controlled	AQ, murders experienced reductions in the physical and verbal aggression
		100% men		1.5 mA 15 min		
		-		3 sessions/consec. days		
**Pathological conditions**						
**Autism spectrum disorders**						
Baruth et al., [67]	25	13.9 ± 5.3	Bilateral DLPFC	rTMS	Randomized-controlled (waiting list)	ABC, reductions in irritability
		84% men and 16% women		1 Hz		
		-		12 sessions 30 min		
				12 sessions/weeks		
Casanova et al., [68]	45	13.0 ± 2.7	Bilateral DLPFC	rTMS	Randomized-controlled (waiting list)	ABC, reductions in irritability
		87% men and 13% women		1 Hz 30 min		
		-		12 sessions/weeks		
Casanova et al., [69]	18	13.1 ± 2.2	Bilateral DLPFC	rTMS	Pre-post design	ABC, reductions in irritability
		78% men and 12% women		0.5 Hz 30 min		
		-		18 weeks/sessions		
Sokhadze et al., [70]	54	14.5 ± 2.9	Bilateral DLPFC	rTMS	Randomized-controlled (waiting list)	ABC, reductions in irritability
		81% men and 19% women		1 Hz 30 min		
		-		18 weeks/sessions		
Wang et al., [71]	33	12.9 ± 3.8	Bilateral DLPFC	rTMS	Pre-post design	ABC, reductions in irritability
		84% men and 16% women		0.5 Hz 30 min		
		-		12 weeks/sessions		
**Unipolar depressed patients of the melancholic subtype (free drugs)**						
Baeken et al., [72]	20	44.3 ± 10.6	Left DLPFC	rTMS active	Single-blind, sham-controlled	POMS, absence differences
		35% men and 65% female		10 Hz 20 min		
		Right-handed		Single session		
**Abstinent smokers**						
Xu et al., [73]	24	45 ± 7.6	Left DLPFC (anodal) and right supraorbital area (cathodal)	tDCS (anodal) 2 mA 20 min	Single-blind, sham-controlled	POMS, absence differences
		87% men and 13% women		Two sessions		
		-				
**Language disorders**						
Andrade et al., [74]	14	From 5 to 12	Anode (Broca area (mid-left inferior frontal gyrus) and cathode right supraorbital area.	tDCS	Pre-post design	35.7% increased irritability: severe (14.3%), moderate (14.3%) and mild (7.1%).
		71% men and 29% women		10 sessions, 2 days interval		
		-				
**Closed-head injury**						
Smith et al., [75]	21	-	Earlobes of patients	CES	Double-blind, sham-controlled	POMS, reductions anger
				100 Hz		
				4 days/week for 3 weeks		
**Alzheimer**						
Wu et al., [76]	52	From 70 to 80	Left DLPFC	rTMS + low dose risperidone	Double-blind, sham-controlled	BEHAVE-AD, reductions aggressiveness
		40% men and 60% women		20 Hz		
		-		5 sessions/week for 4 weeks		
**Refractory partial epilepsy**						
Sun et al., [77]	60	21 years (average)	Epileptogenic	rTMS + antiepileptic drugs (unchanged dose)	Single-blind, sham-controlled	SCL-90-R, absence changes
		68% men and 32% women	focus	0.5 Hz		
		-		Daily for 3 weeks		
**Schizophrenia**						
Hansbauer et al., [78]	146	36 years (average)	Left DLPFC	rTMS	Double-blind, sham-controlled	PANSS, absence changes
		75% men and 25% women		10 Hz		
		82% righ-handed		5 sessions/week for 3 weeks		

Prefrontal cortex (PFC), dorsolateral prefrontal cortex (DLPFC), orbitofrontal cortex (OFC), ventromedial prefrontal cortex (VMPFC), ventrolateral prefrontal cortex (VLPFC), repetitive transcranial magnetic stimulation (rTMS), transcranial direct current stimulation (tDCS), cranial electrotherapy stimulation (CES), theta burst stimulation (TBS), intermittent theta burst stimulation (iTBS), transcranial static magnetic field stimulation (tSMS), State-Trait Anger Scale (STAS), Profile of Mood States (POMS), Visual Analogue Scale (VAS), Beck Depression Inventory (BDI), Buss–Perry Aggression Questionnaire (AQ), Reactive–Proactive Aggression Questionnaire (RPQ), Brunel Mood Scale (BMS), Aberrant Behavior Checklist (ABC), Taylor Aggression Paradigm (TAP), Social Orientation Paradigm (SOP), Behavioral Pathology in Alzheimer’s Disease Rating Scale (BEHAVE-AD), Symptom Checklist-90-R (SCL-90-R), Positive and Negative Syndrome Scale (PANSS).
